# Microencapsulation of *Lactiplantibacillus plantarum* BXM2 in Bamboo Shoot-Derived Nanocellulose Hydrogel to Enhance Its Survivability

**DOI:** 10.3390/gels11060465

**Published:** 2025-06-18

**Authors:** Yajuan Huang, Qiao Guan, Yirui Wu, Chaoyang Zheng, Lingyue Zhong, Wen Xie, Jiaxin Chen, Juqing Huang, Qi Wang, Yafeng Zheng

**Affiliations:** 1 College of Food Science, Fujian Agriculture and Forestry University, Fuzhou 350002, China; hyajuan2022@163.com (Y.H.); qiaofafu1103@163.com (Q.G.); wyr516999@163.com (Y.W.); 18059945008@163.com (C.Z.); xiewen001006@163.com (W.X.); cjx616479284@163.com (J.C.); 2 Institute of Food Science and Technology, Fujian Academy of Agricultural Sciences, Fuzhou 350003, China; lingyue_zhong@163.com (L.Z.); jq_huang@zju.edu.cn (J.H.)

**Keywords:** nanocellulose, probiotics, hydrogel, viability

## Abstract

This study presents a novel approach for enhancing the survivability of *Lactiplantibacillus plantarum* BXM2 using bamboo shoot-derived nanocellulose hydrogels. Nanocellulose hydrogels, composed of cellulose nanofibers (CNFs), cellulose nanocrystals (CNCs), and polyvinyl alcohol (PVA), were developed as protective matrices for probiotics. Fourier transform infrared spectroscopy (FT-IR) and X-ray diffraction (XRD) confirmed the successful formation of hydrogen-bonded networks between PVA and nanocelluloses, while scanning electron microscopy (SEM) revealed that the ternary PVA-CNF-CNC hydrogel exhibited a dense, hierarchical porous structure, effectively encapsulating probiotics with an encapsulation efficiency of 92.56 ± 0.53%. Under simulated gastrointestinal digestion, the encapsulated probiotics maintained 8.04 log CFU/g viability, significantly higher than that of free bacteria (3.54 log CFU/mL). The hydrogel also enhanced heat tolerance (6.58 log CFU/mL at 70 °C) and freeze-drying survival (86.92% viability), outperforming binary systems. During 60-day storage at 4 °C and 25 °C, encapsulated probiotics retained viability above the critical threshold (≥6 log CFU/unit), whereas free cells declined rapidly. These findings highlight the potential of PVA-CNF-CNC hydrogel as an efficient delivery system to improve probiotic stability in food applications.

## 1. Introduction

Probiotics, as a key component of the human gut microbiota, play a crucial role in maintaining and enhancing the balance of intestinal microflora [1]. Adequate intake of live probiotics can promote gut health, protect the intestinal mucosa from pathogens and other harmful microorganisms, and enhance the immune system [2,3,4]. Specific probiotic strains can also modulate the metabolism of nutrients, providing additional benefits such as regulating blood glucose and controlling blood pressure, which are crucial for maintaining overall health [4,5]. However, the viability of probiotics can significantly decrease during food processing, storage, and human digestion, particularly in low-pH gastric acid. The diminished viability could lead to an inadequate quantity of active probiotics arriving in the intestine, which in turn may restrict their capacity to deliver the anticipated health benefits [6,7]. To address this challenge, researchers have proposed the use of encapsulation technology to create a protective coating that shields probiotics from harsh external conditions and enhances their survival in the gastrointestinal tract [8,9,10].

Currently, the materials used for the preparation of probiotic microcapsules can be categorized into pH-responsive materials, specific enzyme-degradable materials, and other macromolecular polymer materials [9]. pH-responsive materials, with alginate being the most popular, are capable of forming a stable alginate gel in acidic environments, which prevents the release of probiotics in stomach acid and thus protects them from gastric acid damage. In the alkaline environment of the intestines, the alginate gel degrades and releases the probiotics, thereby achieving the desired protective effect [11,12]. However, alginate is a porous material, and the microcapsules formed from it have relatively large pores, allowing gastric acid to easily penetrate and damage the probiotics. Additionally, alginate has poor thermal stability, which makes it unsuitable for many food processing requirements. To address the pore defect issue in alginate-based probiotic microcapsules, researchers have proposed the use of specific enzyme-degradable materials, such as chitosan, which can be electrostatically combined with negatively charged polymers to reduce pore size, thereby effectively protecting the probiotics [13,14,15,16]. However, in acidic environments, the repulsion of the NH_3_ groups in chitosan can cause the chitosan gel to swell, leading to the premature release of probiotics in the gastric fluid and therefore reducing the effectiveness of the probiotic microcapsules [17,18]. Pectin is also commonly used as a specific enzyme-degradable material [19,20]. Although it has excellent protective and encapsulation properties, its hydrophilic nature and the protonation of carboxyl groups in gastric acid can cause the pectin gel to swell, leading to the premature release of the encapsulated probiotics [21,22]. Additionally, other macromolecular polymers such as gelatin [23], whey protein [24], polyethylene oxide (PEO) [25], and polyvinyl alcohol (PVA) [26] possess the ability to resist digestion in gastric and intestinal fluids. These polymers can be combined with other materials through electrospinning to prepare probiotic microcapsules [27]. Although electrospinning has been scaled up in some industrial applications (e.g., Bioinicia), the process remains complex and often requires specialized equipment and conditions. Therefore, the discovery of novel materials to address the limitations of traditional microencapsulation materials, enhance the performance of microcapsules, and improve production efficiency and simplicity is a critical challenge for the utilization of probiotics in the food industry.

The evolution of nanomaterial engineering over the past decade has revolutionized probiotic encapsulation strategies, with multifunctional systems demonstrating synergistic protection mechanisms. Advanced wall materials like whey protein isolate (WPI), cellulose nanocrystals (CNCs), and inulin (INU), processed through lyophilization techniques, have been shown to significantly enhance the survival rate of probiotics in the gastrointestinal tract [28]. CNC-reinforced microgels further amplify acid resistance via pore size modulation, reducing gastric fluid penetration [29]. When nanocellulose is combined with other macromolecular substances such as PVA, novel microcapsules can be prepared with outstanding performance in protecting probiotics and promoting their colonization in the gut. Specifically, composite fiber materials formed from nanocellulose fibers (CNFs) extracted from oil palm fruit shells and PVA have been shown to effectively encapsulate probiotics and enhance their stability in the gastrointestinal tract [30]. A novel composite fiber made of cellulose acetate and PVA was utilized to prepare microcapsules via angular dual-nozzle electrospinning, aiming to enhance the gastrointestinal stability of probiotics [27]. Building on this foundation, our prior work established an efficient nanocellulose production protocol: sequential acid–alkali treatment of bamboo shoot shells removed lignin/hemicellulose to isolate fibrous CNF, followed by controlled sulfuric acid hydrolysis to produce needle-like CNC [31,32]. Due to the replacement of some hydroxyl groups on the surface of nanocellulose with anionic groups after acid treatment, CNF and CNC possess abundant negative charges, endowing them with unique biocompatibility and physicochemical properties. While individual CNC/CNF applications have shown protective benefits, the combinatorial potential of CNC-CNF hybrid hydrogels remains unexplored, particularly their capacity to synergistically integrate CNC’s crystallinity-driven barrier properties with CNF’s 3D network-forming abilities, presenting a critical knowledge gap in designing next-generation probiotic matrices.

Building upon the structural synergies between CNC and CNF, we hypothesized that their integration into PVA matrices through dynamic crosslinking could establish a low-cost and highly efficient probiotic stabilization platform. This study aims to develop and evaluate the efficacy of bamboo shoot-derived nanocellulose hydrogels as a protective matrix for enhancing the survivability of *Lactiplantibacillus plantarum* BXM2 under various stress conditions, including freeze-drying, heat treatment, simulated gastrointestinal digestion, and long-term storage. Our findings will validate the hydrogel’s potential as a multifunctional encapsulation matrix for broad-spectrum probiotic applications, particularly addressing developing markets’ requirements for affordable bioactive stabilization technologies.

## 2. Results and Discussion

### 2.1. Characterization of Nanocellulose Hydrogels

#### 2.1.1. FT-IR Analysis

**Figure 1:**
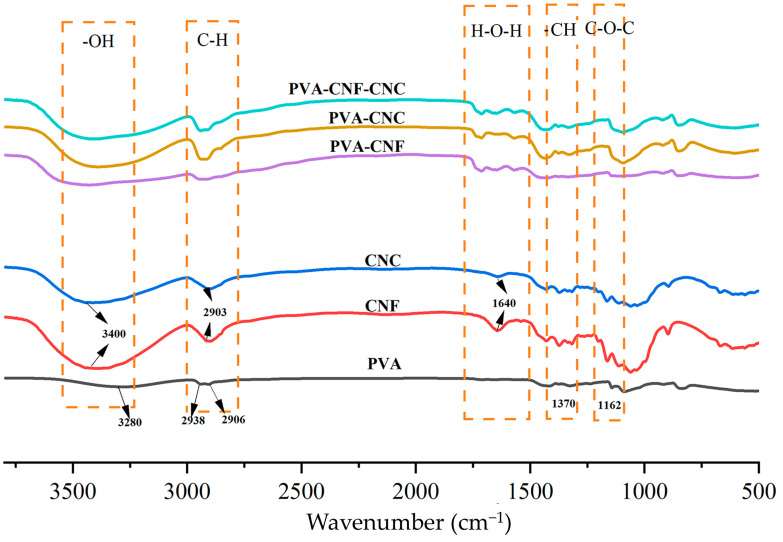
Infrared spectroscopy analysis of PVA, CNF, CNC, and nanocellulose hydrogels.

FT-IR analysis of PVA–nanocellulose hydrogels revealed distinct functional group signatures (Figure 1). The main characteristic peaks of nanocelluloses were observed: 3400 cm⁻^1^ (O-H stretching vibration), 2900 cm⁻^1^ (C-H symmetrical stretching), and 1640 cm⁻^1^ (OH bending of absorbed water) [33,34]. CNF exhibited C-H in plane bending at 1370 cm⁻^1^, while 1162 cm⁻^1^ corresponded to C-O-C/asymmetric ester vibrations. Critical differentiation emerged at 1060 cm⁻^1^, where CNC showed enhanced intensity from superimposed S=O stretching (sulfonic acid groups) and C-O bonds, alongside a prominent 1200 cm⁻^1^ peak confirming sulfate esterification via sulfuric acid treatment [35]. These spectral shifts verify nanocellulose surface modification through hydroxyl substitution with anionic moieties. PVA exhibits several characteristic peaks in infrared spectroscopy that are used for its identification and characterization [36]. One of the main features of PVA is its abundance of hydroxyl (-OH) functional groups. Within the range of 3200–3600 cm^−1^, hydroxyl vibrations appear as broad and intense absorption peaks, providing information on the hydroxyl content and structure of PVA. Additionally, due to the presence of carbon–hydrogen bonds (C-H) from methyl (-CH3) groups in PVA, weaker absorption peaks are observed in the range of 2800–3000 cm^−1^. In the range of approximately 1000–1300 cm^−1^, the main carbon–oxygen bonds (C-O) in PVA can be detected through their absorption peaks, which provide information about ether linkages along the PVA chain [37,38].

FT-IR analysis confirmed hydrogen-bond-mediated interfacial interactions in PVA–nanocellulose composites. In PVA-CNF systems, the O-H stretching vibration redshifted from 3280 to 3420 cm⁻^1^ with concurrent attenuation of the water adsorption peak at 1640 cm⁻^1^, while new signals emerged at 1700/1570 cm⁻^1^, suggestive of carbonyl group reorientation [37,38,39,40]. Similarly, PVA-CNC composites exhibited modified O-H vibrations and crystalline peak broadening at 1200 cm⁻^1^, alongside diagnostic 1700/1570 cm⁻^1^ features. The ternary PVA-CNF-CNC system demonstrated intensified O-H absorption with further redshift, corroborating multi-component hydrogen-bond synergies. These spectral perturbations, particularly the systematic O-H band shifts and emergent carbonyl signatures, quantitatively map hydrogen bond density evolution, while retained cellulose Iβ crystallinity patterns (characteristic 1428/897 cm⁻^1^ peaks) verify interfacial interaction dominance over bulk structural reorganization.

#### 2.1.2. XRD Analysis

**Figure 2:**
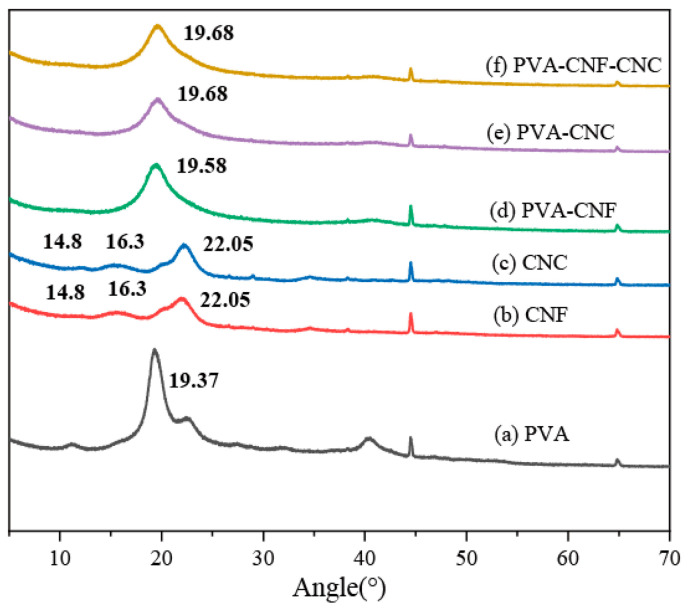
XRD analysis curves of PVA, CNF, CNC, and nanocellulose hydrogels.

XRD analysis revealed structural evolution in nanocomposites (Figure 2). PVA exhibited a monoclinic (101) peak at 19.37° (2θ), while CNF/CNC displayed cellulose Iβ signatures at 14.8°, 16.3°, and 22.05° [39]. Composite systems showed complete nanocellulose peak suppression with PVA peak shifts to 19.58–19.68°, indicating lattice distortion through hydroxyl-mediated interfacial interactions between PVA chains and nanocellulose surfaces. The merged diffraction broadening confirmed disrupted crystalline packing and amorphous phase development.

#### 2.1.3. Morphology of Nanocellulose Hydrogels

**Figure 3:**
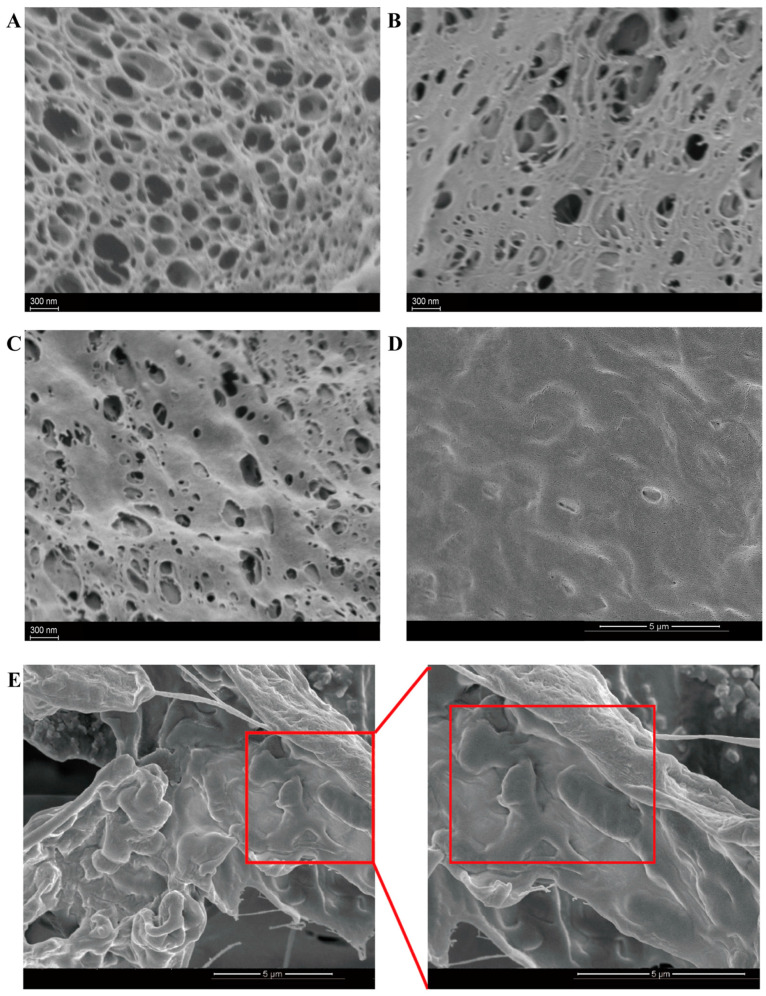
SEM images of nanocellulose hydrogels: (**A**) PVA-CNF; (**B**) PVA-CNC; (**C**) PVA-CNF-CNC; (**D**) surface morphology of PVA-CNF-CNC hydrogel loaded with probiotic; (**E**) cross-section morphology of PVA-CNF-CNC hydrogel loaded with probiotic.

SEM analysis (5000×) revealed nanocellulose-driven architectural evolution: CNF-formed hydrogels displayed lamellar macroporous networks, whereas CNC induced smaller porosity with rod-like nanocrystals, causing aspect ratio-driven packing heterogeneity (Figure 3A,B). The CNC-CNF hybrid system achieved an optimized hierarchical architecture through complementary interactions, demonstrating monodisperse submicron pores, with reduced size variance compared to CNC-only systems (Figure 3C)—a critical feature for gastric acid barrier enhancement. Remarkably, the surface of lyophilized PVA-CNC-CNF probiotics was free of gaps, although slight wrinkling occurred due to freeze-drying, with few free probiotics observed, indicating the effective encapsulation of probiotics in the hydrogel matrix (Figure 3D). Evidenced by 20,000× cross-sectional imaging (Figure 3E), it was demonstrated that a substantial quantity of probiotics was presented within the composite hydrogel. Additionally, probiotics were tightly bound to the nanocellulose hydrogel, with the hydrogel forming a dense film on the surface of the probiotics, effectively encapsulating the bacteria within the microcapsules.

### 2.2. Encapsulation Efficiency of Nanocellulose Hydrogels

**Figure 4:**
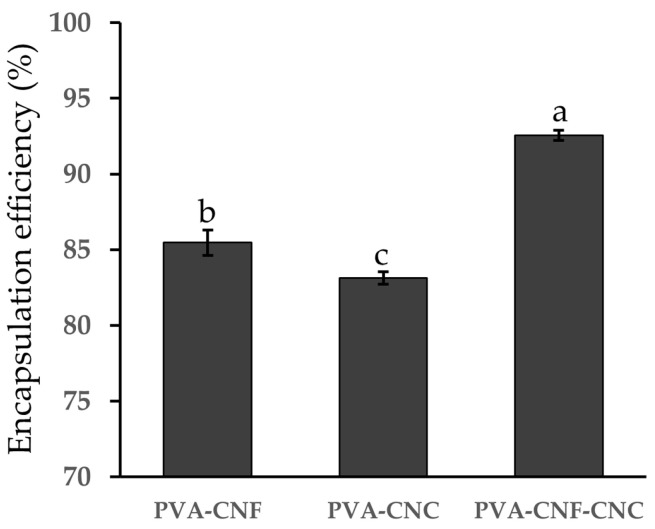
The encapsulation efficiencies of the probiotics of nanocellulose hydrogels. Different letters above the column indicate a significant difference (*p* < 0.05).

Encapsulation efficiency (EE) is a crucial parameter for evaluating both the effectiveness of the encapsulation process and the adequacy of the selected encapsulating agents. As shown in Figure 4, EE analysis demonstrated nanocellulose architectural optimization, with the PVA-CNF-CNC hydrogel achieving a peak EE of 92.56 ± 0.53% for *L. plantarum* BXM2 at an initial loading of 10.03 ± 0.15 log CFU/mL, significantly surpassing binary systems (PVA-CNF: 85.46 ± 0.74%; PVA-CNC: 83.14 ± 0.57%, *p* < 0.05). This high EE was comparable to or even exceeded values reported in other studies. For instance, Pato et al. reported an EE of 90.2% for *Lactobacillus fermentum* InaCC B1295 encapsulated in cellulose microfiber hydrogels derived from oil palm empty fruit bunches (OPEFBs) [30]. Similarly, Sun et al. achieved an EE of 85.6% for *Lactobacillus plantarum* 299 v encapsulated with whey proteins by lyophilization [41]. The superior performance of the PVA-CNF-CNC hydrogel can be attributed to the refined pore architecture, as verified by SEM analysis (Figure 3), and the enhanced matrix density reinforced by hydrogen bonding. The ternary system’s superior confinement capacity originates from CNC-mediated pore size regulation synergizing with CNF’s nanofiber entanglement, creating size-exclusion barriers against bacterial leakage. Consequently, the PVA-CNF-CNC composite hydrogel emerges as a promising candidate for loading *L. plantarum* BXM2. Given its superior performance, this composite hydrogel was selected for further analysis to assess its protective efficiency against various adverse conditions.

### 2.3. Probiotic Viability After Freeze-Drying

**Figure 5:**
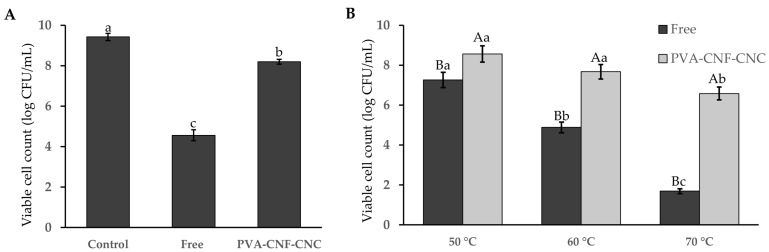
The survivability of probiotics under freeze-drying (**A**) and heat treatments (**B**). Different uppercase letters (A, B) indicate significant differences between samples under the same treatment conditions, while different lowercase letters (a–c) represent significant differences between different treatment conditions for the same samples (*p* < 0.05).

Freeze-drying, while widely utilized for long-term probiotic preservation, imposes critical challenges, including extreme temperature fluctuations, ice crystal formation, dehydration stress, and protein denaturation during processing, which collectively cause mechanical and physiological damage to bacterial cells, drastically reducing their survival rates [42,43]. Comparative analysis revealed a sharp decline in free probiotic viability post-freeze-drying (4.56 log CFU/mL, Figure 5A), whereas nanocellulose hydrogel encapsulation preserved cell counts at 8.19 log CFU/mL, demonstrating superior protection for *Lactobacillus plantarum* BXM2. The survival rate surged from 47.83% to 86.92%, suggesting its dual protective mechanism: nanocellulose not only acts as an efficient cryoprotectant to mitigate rapid freezing-induced cellular damage but also forms a densely compacted structural barrier that shields probiotics from environmental stresses. This synergistic approach significantly enhances both the structural integrity and functional stability of the encapsulated probiotics. Our result is consistent with the findings of Pato et al., who reported that encapsulated *Lactobacillus fermentum* InaCC B1295 retained higher viability (9.09–9.11 log CFU/g) after 28 days of storage at 4 °C compared to free cells [30].

### 2.4. Probiotic Viability After Heat Treatments

Incorporating probiotic bacteria into commercial food products remains a formidable challenge, given that these living microorganisms are exceptionally sensitive to the high temperatures encountered during processing and storage. To evaluate the heat tolerance of encapsulated *Lactiplantibacillus plantarum* BXM2 cells, both free and encapsulated bacteria were subjected to hot water baths for 5 min at temperatures of 50 °C, 60 °C, and 70 °C, respectively. As illustrated in Figure 5B, a significant loss of cell viability was observed in the group of free bacteria following the heat treatments. Concurrently, an enhancement in the heat tolerance of the encapsulated cells was evidenced. When the temperature increased to 70 °C, only 1.69 log CFU/mL of the free bacteria survived. Under the same conditions, the heat resistance of the encapsulated bacteria markedly improved, with a viable cell count of 6.58 log CFU/mL (*p* < 0.05). This result is in line with the study by Sun et al., who reported that encapsulated *Lactobacillus plantarum* 299 v in whey protein hydrogels maintained higher viability during heat treatment [41]. These results highlight the effectiveness of nanocellulose hydrogel microcapsules in protecting probiotic bacteria from thermal damage. The protective mechanism can be attributed to the dense and compact structure of the hydrogel, which effectively isolates the probiotics from heat exposure or at least mitigates its impact.

### 2.5. Probiotic Viability During the In Vitro Digestion Process

**Figure 6:**
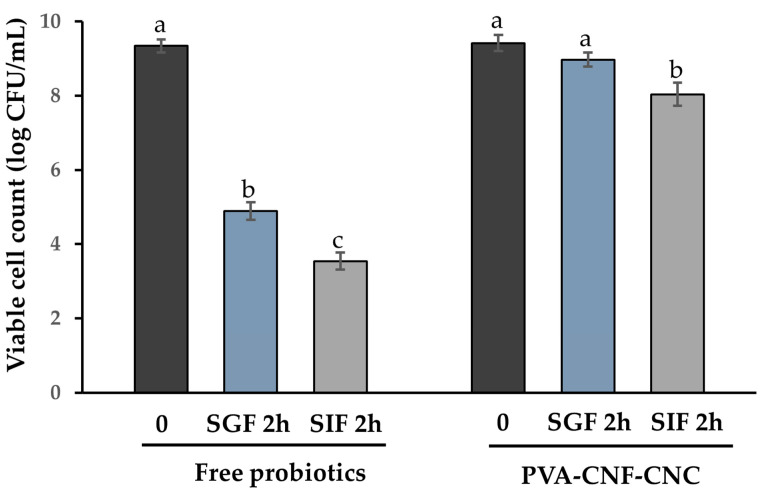
The viability of free and PVA-CNF-CNC-encapsulated *L. plantarum* BXM2 during in vitro digestion simulation. SGF 2 h = two hours of simulated gastric juice digestion; SIF 2 h = two hours of simulated intestinal juice digestion. Different lowercase letters (a–c) indicate significant differences among different sampling times (*p* < 0.05).

In vitro simulated gastrointestinal digestion constitutes an efficacious approach for evaluating the proficiency of probiotic delivery systems [44]. This methodology enables the preliminary evaluation of the capacity of microcapsules to sustain stable probiotic activity. The nanocellulose microcapsules encapsulating probiotics were subjected to a simulated digestion process, wherein initial viable counts were assessed and compared to those of free bacteria following gastric digestion and subsequently intestinal digestion. The results revealed a precipitous decline in the viability of free bacteria, ranging from 9.34 log CFU/mL to 4.89 log CFU/mL after gastric digestion, with a further decrease to 3.54 log CFU/mL after intestinal digestion (as illustrated in Figure 6). These findings underscore the fragility of the microorganisms under such conditions, thereby substantiating the necessity of encapsulation to enhance probiotic survival. Conversely, the probiotic populations within the microcapsules were well-preserved, with 8.04 log CFU/g of viable cells persisting after exposure to simulated gastrointestinal fluids. These outcomes corroborate the efficacy of the nanocellulose hydrogel microcapsule in shielding probiotics from the digestive processes and ensuring the delivery of an adequate quantity of viable probiotics to the intestine to elicit health benefits. Comparatively, the chitosan/casein encapsulation method, as reported by Allahverdi et al., also showed the enhanced survival of encapsulated *Lactiplantibacillus plantarum* in simulated digestive conditions, with viabilities of 6.99 ± 0.12 log CFU/mL in SGF and 7.25 ± 0.23 log CFU/mL in SIJ after 2 h of incubation [45]. The enhanced survival of encapsulated probiotics in simulated gastrointestinal conditions suggests that the PVA-CNF-CNC hydrogel is a promising candidate for improving probiotic stability in food applications. Its ability to maintain high viability levels ensures that an adequate number of active probiotics reach the intestine, where they can exert their intended health benefits.

### 2.6. Probiotic Viability During Storage

**Figure 7:**
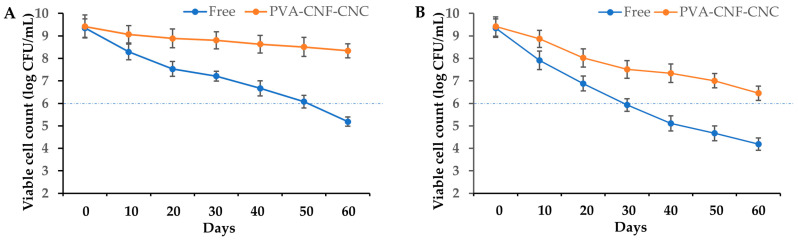
The viability of free and PVA-CNF-CNC-encapsulated *L. plantarum* BXM2 during 60-day storage at 4 °C (**A**) and 25 °C (**B**).

The survivability of probiotics in the food matrix during storage is critically important in industrial applications, with a minimum viable cell count threshold of ≥6 log CFU/unit required at consumption. As illustrated in Figure 7, comparative analysis of *L. plantarum* BXM2 viability revealed substantial differences between PVA-CNF-CNC-encapsulated and free-cell formulations under controlled storage conditions. When stored at 4 °C, free cells exhibited progressive viability loss from 9.35 log CFU/mL to sub-threshold levels (6.08 log CFU/mL at day 50; 5.19 log CFU/mL at day 60), whereas encapsulated counter-parts maintained superior stability with only marginal reduction (8.34 log CFU/mL after 60 days). Elevated temperature testing (25 °C, simulating transport/storage stresses) further accentuated this disparity: free cells degraded below the critical threshold (<6 log CFU/mL) within 30 days, while encapsulated cells retained viability above specifications (6.45 log CFU/g at day 60). These results suggest that encapsulated probiotics possess outstanding storage stability at 4 °C and can tolerate room temperatures for more than 60 days during storage. This enhanced protection can be explained by two aspects. Firstly, the crosslinking of PVA, CNF, and CNC can form a denser layered network structure with uniform small pores, which effectively shields probiotics from external environmental factors, such as oxidative and thermal stressors. Secondly, the nanocelluloses presented in the hydrogel potentially act as the sustained nutrient reservoirs for probiotics. Previous research has also found that under the protection of microcapsules containing fructooligosaccharide and fish oil, the viability of *Lactiplantibacillus plantarum* GIM1.648 slightly declined from 8.82 to 7.98 log CFU/mL after 60 days of storage at 4 °C [46]. In another study, a double layer of microcapsules (alginate/whey protein matrix with CNC outer coating) significantly enhanced the viability of *Lactobacillus casei* LC2W during 120 days of storage, maintaining 6.72 and 5.97 log CFU/g at 4 °C and 25 °C, respectively, after 120 days of storage [47].

## 3. Conclusions

In conclusion, the PVA-CNF-CNC hydrogel developed in this study effectively enhanced the survivability of *Lactiplantibacillus plantarum* BXM2 under various stress conditions. The hydrogel’s dense network structure and hierarchical porosity provided significant protection against environmental stresses, ensuring high viability during freeze-drying, heat treatment, simulated gastrointestinal digestion, and long-term storage. Future work should focus on conducting in vivo studies to validate the probiotic delivery efficiency and health benefits of the hydrogel system. Optimizing the encapsulation process for industrial scalability and continuous production is essential to translate this laboratory-scale success into industrial applications. Exploring the hydrogel’s compatibility with diverse probiotic strains and food formulations, as well as investigating its potential for controlled drug delivery or other biomedical applications, could further expand its utility.

## 4. Materials and Methods

### 4.1. Materials

Bamboo shoot cellulose nanofiber (CNF) and cellulose nanocrystal (CNC) were prepared in our laboratory, as described in previous studies [31,32]. *Lactiplantibacillus plantarum* BXM2 (CGMCC 16436) was graciously provided by the Institute of Food Science and Technology, Fujian Academy of Agricultural Sciences (Fuzhou, China). Polyvinyl alcohol (PVA; CAS: 9002-89-5) was purchased from Aladdin, Shanghai, China. Simulated gastric fluid (SGF) and simulated intestinal fluid (SIF) were purchased from XinFan Biotechnology (Shanghai, China). All other chemical reagents were purchased from Sinopharm Group Chemical reagent Co. (Shanghai, China).

### 4.2. Preparation of Bacterial Suspension

*L. plantarum* BXM2 preserved at −80 °C was thawed and activated by culturing at 37 °C for 24–48 h. Following three generations of subculturing, an appropriate amount of the third-generation bacterial culture was taken and centrifuged at 5500 rpm for 15 min to remove the culture medium. Subsequently, the bacterial pellet was washed twice with sterile physiological saline through centrifugation and then resuspended in sterile physiological saline. The bacterial suspension was diluted to 10^6^-fold and spread on the culture medium for calculation to determine the initial viable cell count (N_0_).

### 4.3. Production of Nanocellulose Hydrogels

The nanocellulose hydrogels were prepared according to Pato et al. [30], with some adjustments, which were a result of previous optimization. First, PVA was dissolved in a water bath at 90 °C to prepare a PVA solution (4% *w*/*v*). After that, CNF (0.5% *w*/*v*) and CNC (5.0% *w*/*v*) were mixed with 10 mL of the PVA solution and heated in a water bath until completely dissolved, resulting in PVA-CNF, PVA-CNC, and PVA-CNF-CNC suspensions. The prepared suspension was uniformly dispersed under the action of a magnetic stirrer and further subjected to ultrasonication for 2 h to ensure thorough the dispersion of CNC and CNF, facilitating the subsequent encapsulation of probiotics. The suspensions were then subjected to high-pressure sterilization at 121 °C for 15 min. The sterile nanocellulose hydrogels were further characterized and subsequently applied as a sealant for probiotics.

### 4.4. Production of Nanocellulose Hydrogels Loaded with Probiotics

The freshly prepared PVA-CNF-CNC suspension was mixed with the bacterial suspension at a 1:1 (*v*/*v*) ratio. A vortex oscillator was employed to thoroughly disperse the bacterial cells evenly throughout the PVA-CNF-CNC matrix. Following incubation at 37 °C on a shaking bed for 1 h, the mixture was frozen at −80 °C for 12 h and then subjected to freeze-drying for 24 h to obtain lyophilized hydrogel loaded with probiotics.

### 4.5. Characterization of Nanocellulose Hydrogels

#### 4.5.1. Fourier Transform Infrared Spectroscopy (FT-IR)

A sample weighing 2.0 mg was placed in an agate mortar, and 100 mg of dry potassium bromide powder was added for grinding. After grinding, the powder was placed in a pressing device to form a transparent film with specific diameter and thickness. The film was analyzed using a Fourier transform infrared spectrometer (Nicolet 5700, Thermo Scientific, Waltham, MA, USA). The analysis of functional groups in nanocellulose hydrogel microcapsules was performed using absorption spectroscopy in the infrared region of 400 to 4000 cm^−1^, with a resolution of 4 cm^−1^ and 16 scans.

#### 4.5.2. X-Ray Diffraction (XRD) Analysis

X-ray diffraction (D8 Advance, BRUKER, Berlin, Germany) was used to determine the crystallinity of the samples and analyze their patterns through wide-angle X-ray diffraction. The test conditions for this experiment were CuKa radiation (λ = 1.5405 × 10^−10^ m), a voltage of 40 kV, a current of 30 mA, a scanning range of 2θ from 5 to 70°, and a scanning rate of 0.05°/10 s. Prior to testing, the samples were ground into powder and thoroughly dried.

#### 4.5.3. Scanning Electron Microscope Analysis

Morphological analyses of the surface and microporous structures of nanocellulose hydrogels (PVA-CNF, PVA-CNC, PVA-CNF-CNC) and probiotic-loaded PVA-CNF-CNC hydrogel were performed using a scanning electron microscope (SEM). The samples were freeze-dried and sputter-coated before SEM observation.

### 4.6. Determination of Encapsulation Efficiency

We added 500 mg of hydrogels loaded with probiotics to 5 mL of 2% sterile sodium citrate solution, adjusted the pH to 6.0, and stirred magnetically at room temperature for 10 min [48]. Subsequently, we diluted the sample with sterile physiological saline, cultured on MRS agar medium, and incubated at 37 °C for 48 h. Finally, we counted the number of bacteria (N) using the plate count method. To facilitate comparison, the viable number of bacteria was expressed as log of colony-forming units (CFUs) per milliliter of bacterial suspension (1.0 g in 1 mL):

Encapsulation efficiency (%) = N/N_0_ × 100

where N is the encapsulated viable cell count, and N_0_ is the initial viable cell count.

### 4.7. Protective Effects of PVA-CNF-CNC Hydrogel

#### 4.7.1. Survival After Freeze-Drying

Referring to the method described by Ashwar et al. [49], 0.1 g of lyophilized hydrogel loaded with probiotics was mixed with 9.9 mL of physiological saline. Subsequently, a 10^6^-fold gradient dilution was conducted on the solution. Next, 0.1 mL of the diluted solution was spread on MRS solid culture medium. The total number of colonies was calculated following a 48 h culture at 37 °C. We compared the results with the sample without lyophilization as a control group for analysis.

#### 4.7.2. Resistance to Heat Treatments

Next, 1 g of sample was mixed with 9 mL of phosphate buffer solution. The mixture was placed in a water bath at respective temperatures of 50 °C, 60 °C, and 70 °C for 5 min. Afterwards, vigorous vortexing was performed to ensure complete dispersion, followed by colony counting. Simultaneously, a 1 mL aliquot of concentrated bacterial suspension was used as a control experiment.

#### 4.7.3. Survival in Simulated Gastric and Intestinal Digestion

The gastrointestinal tolerance of probiotic formulations was evaluated through a standardized in vitro digestion model adapted from a previous study [44]. The encapsulated probiotic (1.0 g) and free probiotic (1.0 mL) were separately introduced into 9.0 milliliters of simulated gastric fluid (SGF, pH 2.5) to mimic physiological stomach conditions. To ensure the equivalence of 1.0 g of encapsulates to 1.0 mL of free probiotic, we measured the initial viable cell count (N_0_) for both the encapsulated and free probiotics. The initial viable cell count was determined by diluting the samples and plating them on MRS agar medium. The results showed that both the encapsulated and free probiotics had an initial viable cell count of approximately 9.3 log CFU/mL, ensuring that the starting conditions were comparable. Following 2 h of gastric phase incubation under constant agitation (150 rpm), the digestive challenge was extended by adding 10 mL of simulated intestinal fluid (SIF, pH 7.0) for a subsequent 2 h intestinal phase simulation. Three critical sampling points were established: baseline (0 h, predigestion), post-gastric digestion (2 h), and complete gastrointestinal transit (4 h). The samples were diluted serially on MRS agar, followed by anaerobic incubation at 37 °C for 48 h. Finally, the viable cells were calculated using automated colony counters, with viability expressed as log CFU/mL through quantitative analysis.

#### 4.7.4. Storage Stability Analysis

To evaluate the storage stability of probiotic formulations, a 60-day comparative study was conducted on free and encapsulated probiotics under controlled temperature conditions (4 °C and 25 °C). The experimental protocol was adapted from an established methodology [47] with specific modifications to simulate practical storage conditions. Samples were suspended in 2 mL of sterile sodium chloride solution within sealed centrifuge tubes, replicating the aqueous environment of liquid-based commercial products. A viability assessment was performed weekly through standardized plate counting techniques to monitor colony-forming units (CFUs). All test groups exhibited an initial bacterial concentration of approximately 9.4 log CFU/mL, ensuring baseline consistency across experimental conditions.

### 4.8. Statistical Analysis

All investigations were conducted in three independent experimental batches (n = 3), with quantitative data presented as mean values ± standard deviation. IBM SPSS Statistics (version 26.0, IBM Corporation, Armonk, NY, USA) was used for statistical analysis, and the significant differences (*p* < 0.05) among the groups were determined by one-way analysis of variance (ANOVA) and Duncan’s multiple comparison tests.

## Data Availability

The original contributions presented in this study are included in this article; further inquiries can be directed to the corresponding authors.
